# Main results of the Korea National Hospital Discharge In-depth Injury Survey, 2004-2016

**DOI:** 10.4178/epih.e2020044

**Published:** 2020-06-20

**Authors:** Sung Ok Hong, Boae Kim, Joongho Jo, Yunhyung Kwon, Yeon-Kyeng Lee, Youngtaek Kim

**Affiliations:** 1 Division of Chronic Disease Control, Korea Centers for Disease Control and Prevention, Cheongju, Korea; 2 Occupational Health Research Department, Korea Occupational Safety and Health Agency, Ulsan, Korea; 3 Public Health and Medical Service Office, Chungnam National University Hospital, Daejeon, Korea

**Keywords:** Injury, Hospitalization, Discharge, Surveillance, Korea

## Abstract

**OBJECTIVES:**

The purpose of this study was to estimate the incidence of injuries and to identify their causes by classifying injuries according to various categories including age, sex, mechanism of injury, body parts injured, and place of injury.

**METHODS:**

This study used data from the Korea National Hospital Discharge In-depth Injury Survey (KNHDIS) from 2004 to 2016. The KNHDIS is conducted annually by the Korea Centers for Disease Control and Prevention, and its survey population includes all hospitalized patients discharged from medical institutions that have 100 or more beds, such as hospitals, general hospitals, and secondary community health centers. The number of injured cases is weighted and estimated using the mid-year estimated population of each year.

**RESULTS:**

The injury discharge rate steadily increased since 2004 (1,505 per 100,000 population in 2004, 2,007 per 100,000 population in 2016) and most injuries were unintentional (annual average of 94.7%). On average, during the 13-year study period, the injury rate for males was 1.5 times as high as for females. The 2 main causes of injury were consistently traffic accidents and falls. Notably, the rate of injuries resulting from falls rose by 1.7-fold from 463 to 792 per 100,000 people, and exceeded the rate of traffic accidents in 2016.

**CONCLUSIONS:**

The incidence of injuries steadily increased after the survey was first conducted, whereas mortality resulting from injuries mostly remained unchanged. This suggests that effective strategies and interventions should be reinforced to reduce unintentional injuries.

## INTRODUCTION

An injury is defined as intentional or unintentional physical and/or psychological damage resulting from an external cause, including conditions such as trauma and poisoning [1]. The World Health Organization classifies injuries as a non-communicable disease (a category that also includes chronic diseases) because injuries follow a chronic course until a previously normal status is restored and often result in residual psychological or biological sequelae. Injuries occur in various circumstances such as violence in schools, industrial disasters, road accidents, agricultural accidents, and accidents at festivals. Injuries are handled by diverse social safety sectors depending on where they occur and whether they are intentional; therefore, there is a need to unify dispersed data to obtain a holistic overview of the epidemiology of injuries.

Korea is ranked as one of the top countries with regard to mortality resulting from injuries [2]. The situations regarding traffic accidents involving children and mortality from industrial accidents were similar to each other, and even though Korea’s safetyrelated indices greatly improved after it joined the Organization for Economic Cooperation and Development (OECD), it still failed to reach OECD standards [3,4].

An analysis of injury-related disease codes in 2016 revealed that the direct health expenditures on injuries covered by national health insurance amounted to 4.0 trillion Korean won (KRW; 2.2 trillion KRW on admissions, 1.8 trillion KRW on outpatients) which accounted for 10% of all medical costs. This reflected a 3-fold increase in injury-related health expenditures since 2004, when expenditures on injuries totaled 1.4 trillion KRW (0.7 trillion KRW on admissions, 0.7 trillion KRW on outpatients) [5].

The social cost due to road accidents, one of the major causes of unintentional injuries, was 26.6 trillion KRW (1.9% of gross domestic product [GDP]) in 2014 [6]. This was a 9.5% increase from the 24.0 trillion KRW spent in 2013 and approximately triple the 9.7 trillion KRW (1.1% of GDP) spent in 2006 [7,8]. The loss of working days due to industrial disasters in 2016 was 47,040, and the estimated economic losses amounted to 21.4 trillion KRW. This was a 4.9% increase from the estimated economic loss of 20.4 trillion KRW in 2015 and double the estimated economic loss of 10.1 trillion KRW in 2002 [9]. It has been estimated that for every 600 near-miss cases of injury, there are 30 cases of property damage, 10 mild injuries, and 1 major injury (the 1-10-30-600 accident ratio) [10,11]. This suggests that the total costs of injuries would be much greater if unidentified injuries were included.

The Korea Centers for Disease Control and Prevention developed the Korea National Hospital Discharge In-depth Injury Survey (KNHDIS), a national representative survey of injury-related discharges from general hospitals in 2005 to understand the scale of injuries, to identify risk factors, and to provide data supporting prevention policies and intervention strategies. On the basis of this nationally representative injury survey, the total number of injuries severe enough to require admission can be estimated. We investigated trends from 2004 to 2016 and reported updated national injury incidence estimates in Korea since 2004.

## MATERIALS AND METHODS

The KNHIDS, which has been conducted since 2004, collects data on approximately 9% of discharged cases from medical institutions with 100 or more beds, including hospitals, general hospitals, and secondary community health centers. Hospitals with a single specialty, long-term care hospitals, geriatric hospitals, military hospitals, and rehabilitation hospitals were excluded even if they had 100 or more beds. The KNHDIS applied stratified 2-stage cluster sampling, with individual hospitals as the primary sampling unit and discharged patients in a sampled hospital as the secondary sampling unit. The hospitals were sampled based on clusters of hospitals stratified by geographic location and number of beds. The hospitals were divided into 4 categories according to the number of beds (100-299, 300-499, 500-999, and over 1,000 beds). Data were collected on patients’ age, sex, residence zip code, type of insurance, diagnostic code based on the International Classification of Diseases 10th revision, hospital admission date and discharge date, and injury-related codes such as the mechanism and place of injury occurrence based on the International Classification of External Causes of Injuries version 1.2.

The KNHDIS data captured multiple admissions with no distinction between initial admissions and readmissions. Approximately 230,000 hospitalizations, including multiple admissions, were reviewed every year, from which 30,000 injury cases were identified and examined. The survey was a complex sample survey, and appropriate weights were applied for the estimation. The number of injured cases was estimated using the mid-year estimated population of each year. Linear variance estimation was carried out using the Taylor series, with the SURVEYMEANS procedure provided by SAS version 9.4 (SAS Institute Inc., Cary, NC, USA) [12].

### Ethics statements

The study was exempt from institutional review board approval as the KNHIDS was conducted a part of national injury surveillance system and all analyses in this study were used public-open data.

## RESULTS

In 2016, 170 sample hospitals were selected out of 561 hospitals, and the estimated total number of discharged injury cases was 1,170,713. This corresponded to 16.5% of the total of discharges for all causes. The injury discharge rate (per 100,000 population) for males was 1.5 times as high as for females during the surveyed period (1.4 to 1.6). Furthermore, the injury discharge rate showed a linear increase, from 1,505 per 100,000 population in 2004 to 2,007 per 100,000 population in 2016.

The injury discharge rate increased with age, and this trend was stable over time. However, the difference in the discharge rate between children aged under 12 years old and people aged over 65 years old in each year widened, from 3.7-fold in 2004 (897 vs. 3,360 per 100,000 population) to 6.5-fold in 2016 (750 vs. 4,887 per 100,000 population). In 2016, patients aged 0-12 displayed the lowest rate (750 per 100,000 population), while the age group of 65 years old and over had a 6.5 times higher value of 4,887. Most injuries were unintentional (93.1% in 2004, 96.4% in 2016) over this period. The traffic accident rate peaked at 772 people per 100,000 people in 2010, and the discharge rate for injuries caused by traffic accidents reached 669 people per 100,000 in 2016, similar to the rate in 2004. The major causes of unintentional injuries were falls (39.4%), traffic accidents (33.4%), and struck by/against injuries (9.7%) in 2016, whereas the major causes in 2004 were traffic accidents (44.5%), falls (30.6%), and struck by/against injuries (6.8%). Furthermore, the discharge rate due to assault decreased by 54.8% from 73 per 100,000 population in 2004 to 40 per 100,000 population in 2016, while that of self-harm injuries remained mostly unchanged (38 per 100,000 people in 2004, 34 per 100,000 people in 2016) (Table 1).

In the 0-12 age group, the number of injury discharges decreased by 59.3% from 67,800 in 2004 to 40,234 in 2016 due to a significant decline of traffic accidents (8,642 in 2016, 26,705 in 2004), which resulted in a sharp drop in roadway injuries.

The number of injuries in the adolescent age group (13-18 years old) and young adults/middle-aged group (19-64 years old) increased by 1.4-fold and 1.2-fold from 2004 to 2016, respectively.

In the elderly group (aged 65 and over), the number of discharged injury cases was 298,208 in 2016, a 1.9-fold increase from 2004. The 2 main causes of injuries were falls (52.4% in 2004, 57.5% in 2016) and traffic accidents (26.7% in 2004, 21.0% in 2016) over this period, although the most dramatic increase was found for struck by/against injuries, the rate of which was 3 times higher in 2016 than in 2004.

Injuries from fractures declined in children (under 12 years old), but rose in older age groups; in particular, in the elderly group (aged 65 and over) the number of injuries from fractures increased by more than 3 times, from 68,370 to 182,455 (Table 2).

## DISCUSSION

Injuries are the leading cause of death in young people aged 10-30 years old, and play a major role in causing premature death at all ages [13]. To establish sustainable development and to reach the standards of developed countries, the rising trend of the incidence of injuries needs to be reversed through safety promotion initiatives. The total incidence of injuries steadily increased since 2004 in this study. Despite the increased incidence of injuries over the last 10 years, the mortality rate due to injuries has remained stable. This suggests that preventative measures against injuries have not been as effective as measures that address their aftermath. A marked increase in injuries in the elderly has resulted in growing medical costs for chronic diseases, with negative impacts on quality of life; as such, injuries in the elderly have the potential to become a huge obstacle to health promotion in Korean society, which is now in an advanced-age era. On the one hand, there have been improvements in survival and functional recovery after serious injuries due to advances in the treatment of emergencies and in the medical system. On the other hand, close attention needs to be paid in order to reduce the incidence of injuries by reinforcing preventative activities that target what happens before accidents and to yield a synergistic effect with health policies aimed at dealing with what happens subsequent to accidents. Furthermore, according to recent study, regional variations need to be prioritized with individual variations such as age and sex [14].

There are several limitations to this study. First, the KNHDIS captured injuries in patients admitted to hospitals with over 100 beds because the KNHIDS was designed to assess trends at the national level, and detailed information on injuries that were recorded as unknown was not identified. As community and individual risk factors were found to influence the risk of hospitalization for unintentional injuries [14], the surveillance system will be considered as a way to collect data at the community level and on geographical characteristics for developing intervention strategies and implementing prevention programs. Although this was a comparative study over time, we utilized the crude rates in the published statistics of the KNHIDS; thus, future research should use age-adjusted data. Despite these limitations, this survey data allowed us to estimate the number of injury cases on the national scale according to injury mechanism, so the present findings have value for utilization in policy-making to prevent injuries. Further comparative research is needed to identify relevant risk factors to reduce unintentional injuries, drawing upon linkage with various sources of health data such as the Korea National Health and Nutrition Examination Survey.

## Figures and Tables

**Table 1.** Estimated injury discharge rates, 2004-2016

Characteristics	2004	2005	2006	2007	2008	2009	2010	2011	2012	2013	2014	2015	2016
Total (n)	1,773	1,871	1,939	1,956	2,010	2,061	2,241	2,199	2,312	2,301	2,313	2,356	2,285
Sex													
Male	2,182	2,269	2,379	2,368	2,390	2,452	2,638	2,571	2,694	2,639	2,620	2,644	2,512
Female	1,359	1,470	1,495	1,541	1,628	1,667	1,843	1,825	1,929	1,962	2,006	2,068	2,056
Age (yr)													
0-12	897	875	818	825	880	832	858	856	882	867	889	822	750
13-18	961	1,098	1,190	1,257	1,356	1,382	1,463	1,582	1,592	1,490	1,533	1,635	1,471
19-64	1,886	1,986	2,033	2,061	2,052	2,122	2,252	2,193	2,262	2,244	2,197	2,229	2,120
≥65	3,360	3,508	3,776	3,550	3,856	3,820	4,446	4,216	4,615	4,564	4,735	4,784	4,887
Intent													
Unintentional	1,505	1,590	1,622	1,669	1,721	1,762	1,947	1,913	2,021	2,006	2,015	2,070	2,007
Intentional	111	113	114	106	112	106	105	101	92	92	90	82	74
Self-harm	38	39	43	33	40	39	40	39	37	40	37	37	34
Assault	73	74	72	72	71	67	65	62	54	52	53	45	40
Unknown	10	10	15	12	4	4	5	-^1^	6	19	16	17	6
Mechanism													
Traffic accident	669	690	714	746	743	745	772	733	771	729	731	738	670
Falls	463	491	518	504	532	558	654	668	703	748	736	783	792
Struck by/against	164	173	246	243	242	239	238	263	274	248	251	252	231
Poisoning	47	47	61	52	55	58	58	55	57	57	59	53	50
Stabbing	86	141	87	72	77	68	74	67	69	75	62	68	77
Unknown	262	292	189	219	250	257	290	286	301	309	320	319	331

Values are presented as number per 100,000 population.

1 Estimates with relative standard error of less than 5.

**Table 2.** Characteristics of injuries by age group

Characteristics	0-12		13-18		19-64		≥65	
	2004	2016	2004	2016	2004	2016	2004	2016
Total	67,800	40,234	34,424	48,223	557,174	682,614	122,049	298,208
Intention								
Unintentional	67,072	39,934	30,613	44,737	509,023	652,438	116,313	291,179
Self-harm	75^3^	-	413^3^	711	15,125	12,489	2,882	4,146
Assault	578^3^	280^3^	3,311	2,629	29,611	16,035	1,447^3^	1,559
Unknown^1^	75^3^	20^3^	88^3^	146^3^	3,414	1,652	1,408	1,324
Mechanism								
Traffic accident	26,705	8,642	12,194	15,770	250,025	256,178	32,567	62,663
Fall	20,980	16,158	9,819	12,875	127,503	205,226	63,991	171,354
Struck by/against	4,899	5,702	7,603	10,095	62,418	87,964	3,834	14,851
Stabbing	3,209	1,372	2,514	1,789	34,270	32,109	1,505	4,315
Fire/heat/flame	4,662	4,269^3^	-	-	-	-	-	-
Poisoning	-	-	194^3^	667	16,883	16,573	4,821	8,087
Unknown^1^	7,345	4,091	2,101	7,027	66,075	84,564	15,331	36,938
Source of traffic accident								
Subtotal	26,705	8,642	12,194	15,770	250,025	256,178	32,566	62,663
Pedestrian	14,245	3,797	2,415	2,530	31,841	30,631	8,582	16,099
Bicycle	4,295	2,466	1,357	2,964	9,441	22,044	2,907	6,975
Motorcycle	-	-	4,205	5,622	24,776	24,167	5,293	10,449
Automobile	5,672	2,160	3,103	3,817	134,558	152,443	5,612	18,079
Unknown^1^	2,493	219^3^	1,114^3^	837^3^	49,409	26,893	10,172	11,061
Place of injury								
Residence	16,905	8,885	3,337	2,814	58,273	65,448	34,717	78,504
School	5,255	3,331	4,611	4,345	-	-	-	-
Field/stadium	3,840	2,329	3,410	6,172	15,798	20,060	-	-
Roadway	22,754	8,714	12,926	16,312	237,336	270,717	31,501	71,782
Workplace	-	-	-	-	6,361	42,078	-	-
Farm	-	-	-	-	-	-	1,255^3^	10,245
Lake·Steam·sea·outdoor	-	-	-	-	-	-	-	5,816
Unknown^1^	19,046	16,975	10,140	18,580	239,406	284,311	54,576	131,861
							120,794	298,208
Nature of injury								
Fracture	25,166	19,843	15,221	19,379	167,256	235,910	68,370	182,455
Dislocation^2^	231^3^	190^3^	330^3^	888	4,807	8,608	1,371^3^	1,947
Sprain/strain^2^	3,290	2,197	3,756	10,974	127,891	178,330	8,892	23,992
Internal organ	12,813	4,305	6,004	4,356	101,031	77,773	21,291	35,477
Open wound	8,734	2,330	2,715	1,523	36,204	25,589	4,830	9,036
Traumatic amputation	937^3^	108^3^	72^3^	73^3^	10,355^3^	4,656	1,108^3^	534^3^
Vascular injury	15^3^	111^3^	-	140^3^	2,336	2,472	145^3^	527^3^
Contusion·superficial injury	4,638	3,177	2,138	3,738	32,447	42,885	5,796	17,277
Crush injury	1,755^3^	46^3^	206^3^	103^3^	10,388	1,962	682^3^	375^3^
Burn	5,872^3^	4,607^3^	613^3^	879^3^	14,198	19,229^3^	2,106	6,423
Nerve injury	138^3^	259^3^	485^3^	154^3^	2,957	2,992	119^3^	405^3^
Poisoning	723	533	227^3^	668	19,033	15,414	6,749	7,870
Others	4,201	2,494	3,466	5,205	36,620	66,016	4,255	11,728
Unknown^1^	526	34^3^	338^3^	143^3^	4,178	778	218^3^	162^3^

Values are presented as number of estimated cases.

1 Unknown includes unclassified cases.

2 Dislocations, sprains, and strains were collected without a detailed classification.

3 Estimates with relative standard error of 25% or greater.
